# Detecting Digoxin Toxicity by Artificial Intelligence-Assisted Electrocardiography

**DOI:** 10.3390/ijerph18073839

**Published:** 2021-04-06

**Authors:** Da-Wei Chang, Chin-Sheng Lin, Tien-Ping Tsao, Chia-Cheng Lee, Jiann-Torng Chen, Chien-Sung Tsai, Wei-Shiang Lin, Chin Lin

**Affiliations:** 1 Division of Cardiology, Department of Medicine, Tri-Service General Hospital, National Defense Medical Center, Taipei 11490, Taiwan; dwc530@gmail.com (D.-W.C.); littlelincs@gmail.com (C.-S.L.); wslin545@ms27.hinet.net (W.-S.L.); 2 Division of Cardiology, Heart Centre, Cheng Hsin General Hospital, Taipei 112, Taiwan; 9206battery@gmail.com; 3 Planning and Management Office, Tri-Service General Hospital, National Defense Medical Center, Taipei 11490, Taiwan; clee112@mail.ndmctsgh.edu.tw; 4 Division of Colorectal Surgery, Department of Surgery, Tri-Service General Hospital, National Defense Medical Center, Taipei 11490, Taiwan; 5 Department of Ophthalmology, Tri-Service General Hospital, National Defense Medical Center, Taipei 11490, Taiwan; jt66chen@gmail.com; 6 Division of Cardiovascular Surgery, Department of Surgery, Tri-Service General Hospital, National Defense Medical Center, Taipei 11490, Taiwan; sung1500@mail.ndmctsgh.edu.tw; 7 School of Public Health, National Defense Medical Center, Taipei 11490, Taiwan; 8 School of Medicine, National Defense Medical Center, Taipei 11490, Taiwan; 9 Graduate Institute of Life Sciences, National Defense Medical Center, Taipei 11490, Taiwan

**Keywords:** artificial intelligence, electrocardiogram, deep learning algorithm, digoxin toxicity

## Abstract

Although digoxin is important in heart rate control, the utilization of digoxin is declining due to its narrow therapeutic window. Misdiagnosis or delayed diagnosis of digoxin toxicity is common due to the lack of awareness and the time-consuming laboratory work that is involved. Electrocardiography (ECG) may be able to detect potential digoxin toxicity based on characteristic presentations. Our study attempted to develop a deep learning model to detect digoxin toxicity based on ECG manifestations. This study included 61 ECGs from patients with digoxin toxicity and 177,066 ECGs from patients in the emergency room from November 2011 to February 2019. The deep learning algorithm was trained using approximately 80% of ECGs. The other 20% of ECGs were used to validate the performance of the Artificial Intelligence (AI) system and to conduct a human-machine competition. Area under the receiver operating characteristic curve (AUC), sensitivity, and specificity were used to evaluate the performance of ECG interpretation between humans and our deep learning system. The AUCs of our deep learning system for identifying digoxin toxicity were 0.912 and 0.929 in the validation cohort and the human-machine competition, respectively, which reached 84.6% of sensitivity and 94.6% of specificity. Interestingly, the deep learning system using only lead I (AUC = 0.960) was not worse than using complete 12 leads (0.912). Stratified analysis showed that our deep learning system was more applicable to patients with heart failure (HF) and without atrial fibrillation (AF) than those without HF and with AF. Our ECG-based deep learning system provides a high-accuracy, economical, rapid, and accessible way to detect digoxin toxicity, which can be applied as a promising decision supportive system for diagnosing digoxin toxicity in clinical practice.

## 1. Introduction

Digoxin has been widely used for heart failure (HF) and atrial fibrillation (AF) treatment in the past several decades. Guidelines from the American College of Cardiology/American Heart Association and the European Society Cardiology suggest that patients with symptomatic heart failure with reduced ejection fraction (HFrEF), New York Heart Association (NYHA) Functional class II–IV, may consider digoxin to reduce both all-cause and HF-associated hospitalizations in cases of limited response to standard treatment [1,2,3]. Digoxin is also important in acute heart rate control for patients with HF and AF in the absence of pre-excitation, and in resting heart rate control for patients with HFrEF [4]. All the evidence supports the indispensable role of digoxin in clinical practice.

However, digoxin toxicity accounts for 0.3% of all the adverse drug events in the U.S., among which 82.1% were hospitalized due to its narrow therapeutic window [5]. The in-hospital mortality for a non-digoxin immune Fab (DIF) group was 5.7%, while it was 14.3% for a DIF group in patients who were hospitalized for digoxin toxicity [6]. Another descriptive observational study revealed 6.4% immediate and 13.4% 30-day mortality rates among 171 digoxin poisoning patients in the emergency departments (EDs) [7]. For those with digoxin toxicity causing potentially life-threatening hyperkalemia, cardiac rhythm disturbances, or both, early administration of digoxin-specific Fab antibody fragments may improve mortality rate and has a class I indication [8,9,10]. The results highlight critical effects of digoxin toxicity.

Nonetheless, laboratory investigation for serum digoxin concentration (SDC) is time-consuming and might not be available in all hospitals. SDC may be dynamic, which is confounded by several comorbidities, including acute kidney injury, low cardiac output, or concomitant use of other drugs [11]. In fact, the narrowing in the therapeutic to toxicity window, difficult dose adjustment, and poor accessibility of serum concentration have markedly limited the use of digoxin.

The epidemiology of digoxin toxicity accounts for about 1% of patients with congestive HF who are treated with digoxin [12]. The pharmacological effect of digoxin may be manifested by electrocardiography. The electrocardiographic manifestations for digoxin toxicity are well documented, including sinus bradycardia, AF with slow ventricular response, atrioventricular and intraventricular conduction disturbance, down-sloping ST-T alterations (especially at leads V4 to V6, lead II, III, aVF), ST segment elevation in leads aVR and V1, shortening of QT interval, atrial tachycardia with block, junctional tachycardia, ventricular tachycardia, and frequent premature atrial or ventricular beats. A previous retrospective study demonstrated that 39.2% of digoxin toxicity patients’ ECGs are ignored by clinicians [13], and another prospective study revealed that only 23.95% of digoxin toxicity patients’ ECGs are recognized by clinicians as positive findings [14]. These results elucidate the high misdiagnosis and delayed diagnosis of digoxin toxicity in clinical practice. 

Artificial intelligence (AI) has been extensively applied in the biomedical fields, including cardiovascular risk prediction, and it automates diagnosis of eye diseases, reviews pathological slides, and triages urgent findings in head CTs [15,16,17] to promote health care. Moreover, neural networks have been applied to classify the changes in the EEG signal by facial expressions [18]. However, there has been no application of AI in the diagnosis of digoxin toxicity. In this study, we trained a deep learning model (DLM) to use ECG to identify digoxin toxicity and conducted a human-machine competition to verify its performance.

## 2. Materials and Methods

### 2.1. Database

The Tri-Service General Hospital, Taipei, Taiwan, provided the research data from November 2011 to February 2019. Research ethics approval was given by the institutional review board to collect data without individual consent (IRB NO C202005055). Because digoxin toxicity is less likely to occur with SDC < 2 ng/mL, the digoxin toxicity cases were defined as more than or equal to 2 ng/mL in blood concentration based on laboratory testing [19,20]. All ECG within 24 h before or after digoxin toxicity (SDC no less than 2 ng/mL) samples were collected and were reviewed by our cardiologist. There were a total of 36 cases with SDC no less than 2 ng/mL in our database with documented electrocardiography, but 2 of the 36 cases were excluded due to their poor quality, number of artifacts, and deviated baseline ECG. We used ICD-9 and ICD-10 codes to define baseline comorbidities as following: diabetes mellitus (DM, ICD-9 codes 250.x and ICD-10 codes E11.x), coronary artery disease (CAD, ICD-9 codes 410.x to 414.x, and 429.2, and ICD-10 codes I20.x to I25.x), hypertension (HTN, ICD-9 codes 401.x to 404.x and ICD-10 codes I10.x to I16.x), heart failure (HF, ICD-9 codes 428.x, 398.91, and 402.x1, and ICD-10 codes I50.x), lipidemia (ICD-9 codes 272.x and ICD-10 codes E78.x), chronic kidney disease (CKD, ICD-9 codes 585.x and ICD-10 codes N18.x), chronic obstruction pulmonary disease (COPD, ICD-9 codes 490.x to 496.x and ICD-10 codes J44.9), pneumothorax (ICD-9 codes 512.x and ICD-10 codes J93.x), and atrial fibrillation (AF, ICD-9 codes 427.31 and ICD-10 codes I48.x), lipidemia (ICD-9 codes 272.x and ICD-10 codes E78.x). Ultimately, we collected a total of 61 ECGs from 34 cases as the digoxin toxicity samples. Other details and deep learning model implantation are provided in supplementary information.

### 2.2. Deep Learning Model Implement

We developed a deep learning model called ECG12Net to use 12 lead ECGs for potassium concentration prediction. The technology details, such as model architecture, data augmentation, and model visualization, were described previously [21].

ECG12Net is an 82-layer convolutional neural network with ECG lead blocks to extract the important features and an attention mechanism to integrate predictions from each lead. Each ECG lead block used the same weights except the last fully connected layer. We employed the same architecture to train a new deep learning model for digoxin toxicity recognition. The only change in this model is the last layer, which is a logistic output revised from linear output. The settings for the training model were as follows: (1) Adam optimizer with standard parameters (β1 = 0.9 and β2 = 0.999) and a batch size of 50 for optimization; (2) learning rate was set at 0.001; and (3) a weight decay of 10^−4^ [22]. The 100th epoch model was used as the final model, in which the presented performance in the validation set was only evaluated once.

Because the sampling rate of our machine is 500 Hz, our 12-lead ECG signal includes 12 numeral sequences with 5000 digits. However, the standard input format of ECG12Net is a length of 1024 numeric sequences. We randomly cropped a length of 1024 sequences as input in the training process. During the inference stage, the 9 overlapping lengths of 1024 sequences based on interval sampling (X1 to X1024, X498 to X1521, X995 to X2018, X1492 to X2515, X1989 to X3012, X2486 to X3509, X2983 to X4006, X3480 to X4503, and X3977 to X5000) were used to generate predictions and were averaged as the final prediction. (The complete architecture of ECG12Net is shown in Supplementary Figure S1). 

Due to significantly uneven distribution of digoxin toxicity cases and controls in our study, an oversampling process was implemented to ensure that rare samples were adequately recognized [23,24]. The initial weights of the digoxin toxicity recognition model were based on the transfer learning from the potassium concentration prediction model [21]. The patients in the training cohort were divided into 5 subgroups, and we used the 5-fold cross-validation which used 4 of them as a training subset and 1 of them as a tuning subset in each fold. In other words, we totally trained 5 DLMs, and the final prediction in validation cohorts was generated by the average of them. The cut-off point to distinguish the positive sample was also decided via each tuning subset.

### 2.3. Human-Machine Competition

We evaluated the performance of practicing physicians using a subvalidation cohort. All 13 digoxin toxicity ECGs in the validation cohort were included, and 52 normal ECGs in the validation cohort were randomly selected for the human-machine competition. There were 4 emergency physicians and 5 cardiologists participating in this competition. We defined 5 or more of the 9 clinicians’ answers as human answers in the human-machine competition. The disease score of digoxin toxicity, ranging from 0.000 to 1.000, higher than 0.334 was known as high risk for the AI machine.

### 2.4. Statistical Analysis and Model Performance Assessment

All analyses were based on ECGs from the patients. If more than 1 ECG is collected from a single patient, each ECG will be analyzed as independent cases. We presented their characteristics as the means and standard deviations, numbers of patients, or percentages, where appropriate. They were compared using either Student’s *t* test or the chi-square test, as appropriate. We used a significance level of *p* < 0.05 throughout the analysis. The statistical analysis was performed using the software environment R version 3.4.4 (Comprehensive R Archive Network, Vienna, Austria). The software package MXNet version 1.3.0 (The Apache Software Foundation, Wakefield, MA, USA) was implemented into our deep learning model. The primary analysis was to evaluate the performance of digoxin toxicity recognition by ECG12Net and clinicians in a machine-human competition. Receiver operating characteristic (ROC) curve and area under curve (AUC) are applied to evaluate the competition results. Additionally, the sensitivity, specificity, and balance accuracy of digoxin toxicity recognition by the deep learning model and clinical physicians were calculated. The balance accuracy is defined as the mean of sensitivity and specificity in the study. The results of the whole validation evaluation by the DLM are also presented. 

The secondary analyses were performed on the whole validation set. We tried to include more clinical information, such as patient characteristics and laboratory test, to improve the model performance. The multivariable logistic regression model was used to integrate the deep learning model and clinical information. A series of logistic models will identify the effects of different clinical information on the performance of digoxin toxicity recognition. The AUCs based on the ROC curve are applied to evaluate the change of model performance. 

## 3. Results

A total of 177,127 ECGs were enrolled in the study (Supplementary Table S1) and were divided into either a training cohort (*n* = 160,916) or a validation cohort (*n* = 16,211). Further stratified analysis divided the cohort into a digoxin toxicity group and a normal group by ECG by artificial intelligence ECG analysis (Table 1), which demonstrated that patients with digoxin toxicity records were prone to be older and had more comorbidities, such as atrial fibrillation (AF), hypertension, HF, coronary artery disease (CAD), chronic obstructive pulmonary disease (COPD), hyperlipidemia, and chronic kidney disease (CKD), and had higher serum potassium levels, lower serum sodium levels, higher serum blood urea nitrogen levels, higher serum creatinine levels, and worse estimated glomerular filtration.

### 3.1. Human-Machine Competition

**Figure 1:**
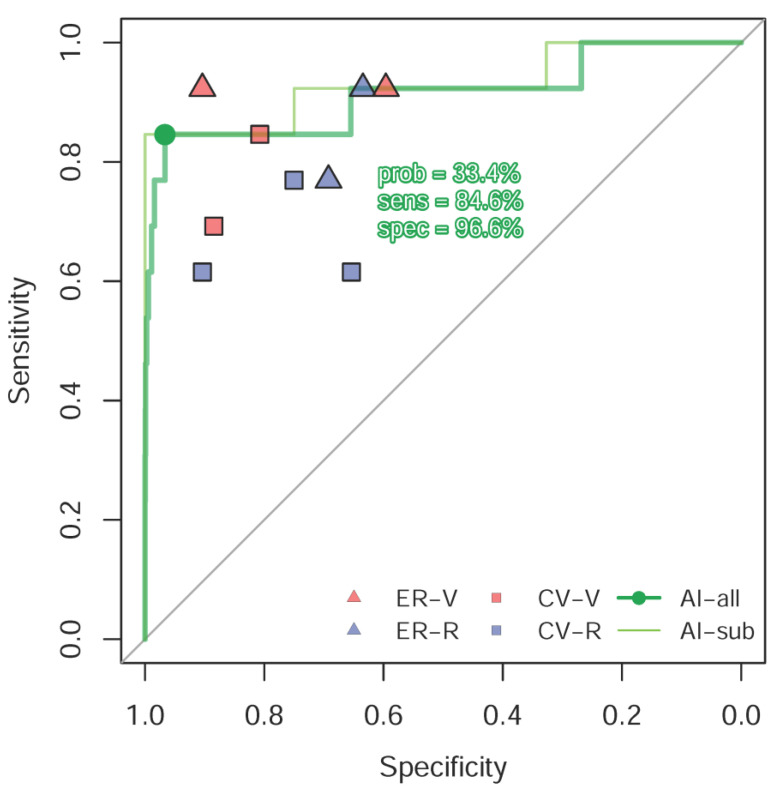
Performance comparison in digoxin toxicity recognition in the human-machine competition. The receiver operating characteristic curves (ROC curves) were made by the predictions of the deep learning model in the validation cohort (AI-all) and the human-machine competition (AI-sub), respectively. The red and blue points represent visiting staff members and residents, respectively. The triangle and square marks represent the emergency physicians and cardiologists, respectively. The areas under curve (AUCs) were 0.912 and 0.929 in the validation cohort and the human-machine competition, respectively. The sensitivity was 84.6%, the specificity was 96.6%, and the probability was 33.4% in the validation cohort. The positive predictive value in the validation cohort was 1.98%, which provided an F1 score of 3.87%. prob = probalility; sens = sensitivity; spec = specificity.

Figure 1 shows the summary results of the human-machine competition, and the ROC curve was made based on the predictions of the deep learning model. The AUC of our AI system in the human-machine competition was 0.929, and only a visiting emergency staff member with five years of experience was superior to our AI system. The AI model was noninferior to a visiting emergency staff member with 14 years of experience, a visiting cardiovascular department staff member with five years of experience, and an emergency chief resident. The AI method was superior to the other participating clinicians. The ROC curve analysis was also conducted in the validation cohort, and the AUC of our AI system was 0.912, which provided a sensitivity of 84.6%, a specificity of 96.6%, a positive predictive value of 1.98%, and an F1 score of 3.87%. The lower positive predictive value and F1 score were due to the rarity of digoxin toxicity cases.

The consistency analysis of the human-machine competition for digoxin toxicity risk prediction by ECG reading is shown in Supplementary Figure S2, which also presents the sensitivities and specificities of each physician. Supplementary Figure S2A shows all digoxin toxicity ECGs, ranking from lower disease score to higher disease score by AI analysis, and the Supplementary Figure S2B shows the selected normal ECGs, ranking from higher disease score to lower disease score. The other 39 normal ECGs in the human-machine competition were consistently considered as negative samples by both our AI system and the human experts. The disease score provided by our AI system was consistent with the humans’ consensus. We divided the results into four quadrants. In the first quadrant, both humans and the machine had correct answers. In the second quadrant, AI had correct answers, but humans did not. In the third quadrant, one case was correctly answered by humans but was missed by the machine, and in the fourth quadrant, only case A was missed by both humans and the machine.

**Figure 2:**
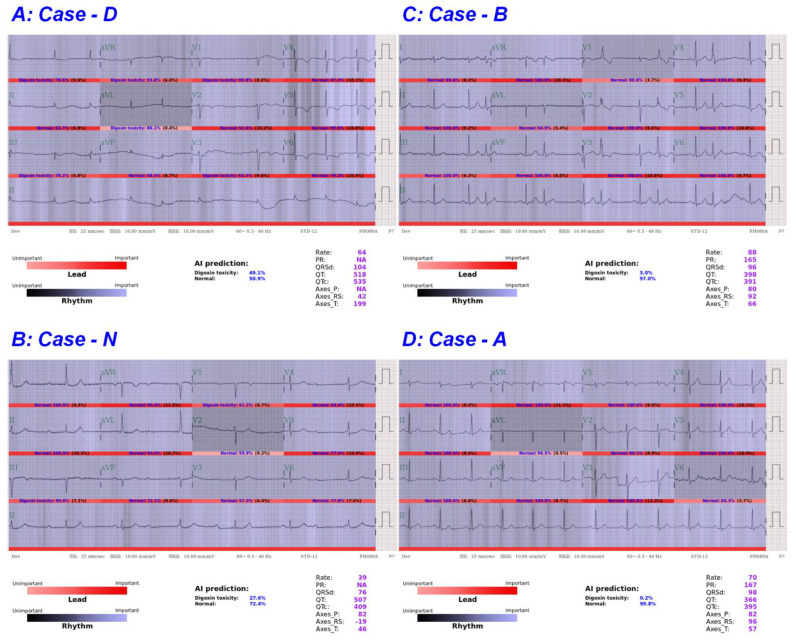
Selected ECGs (electrocardiographies) of consistent and inconsistent assessments given by the deep learning model and the human experts. (**A**) The ECG was correctly identified as high risk of digoxin toxicity by both human experts and AI machine. (**B**) The ECG was correctly identified as low risk of digoxin toxicity by AI machine, but not by human experts. (**C**) The ECG was correctly identified as high risk of digoxin toxicity by human experts, but not by AI machine. (**D**) The ECG has been misdiagnosed by both human experts and AI machine.

ECGs in the first quadrant revealed typical characteristics of digoxin toxicity in high-risk cases, such as concave or down-sloping ST-T depression at the inferior and lateral leads, diminished T wave height, and bradycardia with AF with a slow ventricular response. For example, in case D (Figure 2A), the electrocardiograph revealed AF with a rate of 64 beats per minute and typical down-sloping ST depression from V4 to V6 that was unlikely related to left ventricular hypertrophy (LVH). All of the clinicians and AI identified the case as high risk. The SDC of case D was 2.6 ng/mL. In the second quadrant, there were some nonspecific findings that may have confused the clinicians, such as complete atrioventricular block, junctional bradycardia, down-sloping ST depression due to ischemia, hypokalemia, or other etiologies [25]. In case N (Figure 2B), the electrocardiograph showed complete AV block with 0.5 mm ST depression at leads I, II, and V4 to V6, which was suggested as digoxin toxicity by human experts. The AI revealed negative results for digoxin toxicity from the electrocardiography, which is the correct answer. In the third quadrant, the case B (Figure 2C) electrograph showed sinus rhythm with bigeminy ventricular premature complexes (VPCs), which was identified as digoxin toxicity by human experts but misdiagnosed by the AI machine. In the fourth quadrant, the ECG of case A who had an SDC of 2.5 ng/mL (Figure 2D) revealed a normal sinus rhythm electrocardiograph, which was misdiagnosed by all the clinicians and AI.

### 3.2. Predictive Abilities of the Individual Lead

**Figure 3:**
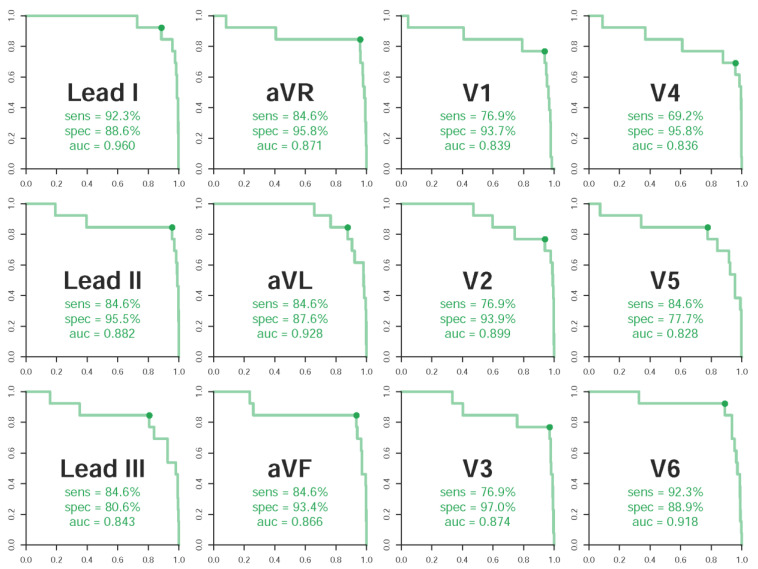
Lead-specific ROC (Receiver operating characteristic) curves in the validation cohort. ROC curves with specificity on the *x*-axis and sensitivity on the *y*-axis. The cases were defined as the digoxin toxicity ECGs, and the controls were defined as the normal ECGs.

We analyzed all the 12 leads’ predictive abilities. The most valuable leads for digoxin toxicity prediction were lead I (sensitivity 92.3%, specificity 88.6%, AUC 0.960), lead aVL (sensitivity 84.6%, specificity 87.6%, AUC 0.928), and V6 (sensitivity 92.3%, specificity 88.9%, AUC 0.918) (Figure 3). These three leads presented better performance compared to the integration of all 12 leads (sensitivity 84.6%, specificity 96.6%, AUC 0.912). However, the summation of sensitivity and specificity in the integration model was not less than the performance of each lead.

### 3.3. Univariable and Multivariable Logistic Regression Analysis

Univariable and multivariable logistic regression analyses on the digoxin toxicity in training cohort are shown in Supplementary Figure S3. Higher SDC is associated with male gender (OR: 2.21, 95% CI: 1.19–4.12, *p*-value: 0.012), older age (OR: 1.09, 95% CI: 1.06–1.12, *p*-value: <0.001), lower Body Mass Index (BMI) (OR: 0.91, 95% CI: 0.83–0.99, *p*-value: 0.038), medical history of CAD (OR: 2.67, 95% CI: 1.51–4.71, *p*-value: 0.001), hypertension (OR: 19.08, 95% CI: 5.93–61.40, *p*-value: <0.001), HF (OR: 7.07, 95% CI: 4.01–12.46, *p*-value: <0.001), hyperlipidemia (OR: 2.99, 95% CI: 1.69–5.29, *p*-value: <0.001), COPD (OR: 3.93, 95% CI: 2.23–6.92, *p*-value: <0.001), AF (OR: 29.00, 95% CI: 15.75–53.41, *p*-value: <0.001), higher serum potassium concentration (OR: 1.99, 95% CI: 1.48–2.69), lower serum sodium concentration (OR: 0.95, 95% CI: 0.90–1.00, *p*-value: 0.046), higher serum level of blood urea nitrogen (BUN) (OR: 1.02, 95% CI: 1.01–1.03, *p*-value: <0.001), higher serum level of creatinine (OR: 1.16, 95% CI: 1.08–1.24, *p*-value: <0.001), and worse estimated Glomerular Filtration Rate (eGFR) (OR: 0.97, 95% CI: 0.96–0.98, *p*-value: <0.001). Because of the higher missing rates of laboratory tests, we conducted a multivariable analysis only for patient demographics and disease histories. Only gender, age, hypertension, and AF had independent effects to identify digoxin toxicity. In the subsequent analysis considering the laboratory tests, eGFR results were the only laboratory data that had an independent effect to identify digoxin toxicity. We attempted to add the predictive ability combined with our AI system (Supplementary Table S2). The integration model’s AUC improved from 0.912 (AI model) to 0.980 (integration model 1) and 0.987 (integration model 2), but there was no statistical significance (*p*-value 0.260, 0.207, respectively).

### 3.4. Diagnostic Capacity in Patients with HF or AF

**Figure 4:**
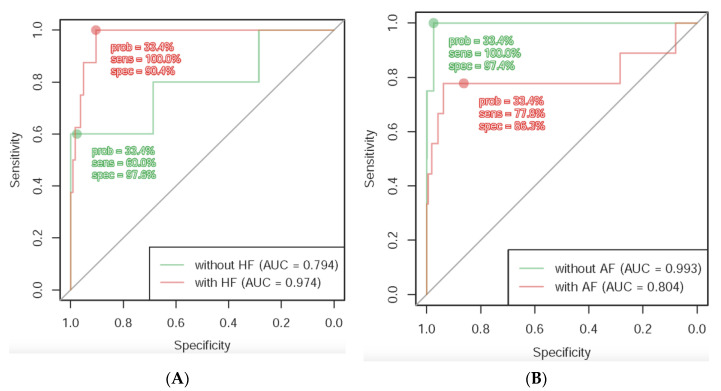
ROC curves under significant stratified variables. (**A**) Better prediction of the AI model may be noted with a larger AUC in patients known to have heart failure; (**B**) Worse prediction of the AI model with smaller AUC for patients with atrial fibrillation.

Because digoxin has class I indications for HF and AF treatment, we analyzed whether HF or AF may interfere with the accuracy of the AI ECG reading. The results are demonstrated in Figure 4. For patients with HF, the sensitivity of AI identification is 100% and the specificity is 90.4%, while for patients without HF, the sensitivity is 60.0% and the specificity is 97.6%. In comparison, for patients with AF, the sensitivity is 77.8% and the specificity is 86.3%, while for patients without AF, the sensitivity is 100.0% and the specificity is 97.4%. The results revealed a larger AUC in patients known to have HF (Figure 4A) but worse AUC for patients with AF (Figure 4B).

## 4. Discussion

Our AI model provides an 84.6% sensitivity and a 96.6% specificity predictive ability in the detection of patients with digoxin toxicity. The human-machine competition showed that the performance of our AI system was not inferior to cardiologists and ER specialists. Interestingly, the performance of the DLM using lead I (AUC = 0.960) was not inferior to that using all 12 leads (AUC = 0.912) of the ECG. The information of HF and AF enhanced the diagnostic power of digoxin toxicity by our DLM.

The mechanisms of digoxin on ECG manifestations were studied. Digoxin affects multiple cellular processes by inhibition of Na(+), K(+)-ATPase (adenosine triphosphatase), which raises the intracellular Ca^2+^ concentration of cardiac myocytes and induces prolongation of cardiac action potential in phase 4 and phase 0, resulting in an increasing AV node refractory period, prolongation of the PR interval, and decrease of ventricular response [26,27]. At serum concentrations of 0.5 to 1.9 ng/mL, digoxin is prone to decrease sympathetic nervous system activity and to augment vagal tone, resulting in maximal diastolic resting membrane potential increase and automatic decreases in atrial and AV nodal cells. These effects are accompanied by effective refractory period prolongation and AV nodal conduction velocity decrease. The shortening of the atrial and ventricular refractory periods may cause a short QT interval with secondary repolarization abnormalities, which affects the ST segments and T waves. In addition, increased vagal effects at the AV node may cause a prolonged PR interval. At a higher SDC, digoxin can increase sympathetic nervous system activity and prolongation of AV conduction, resulting in bradycardia, heart block, or other life-threatening arrhythmias [28].

Previous machine learning studies used several variables including drug-to-drug interaction, demographic items, laboratory items, and medical history to set up optimal SDC prediction models with the AUC ranging from 0.533 to 0.912 [29,30]. Importantly, our deep learning model predicted high-risk digoxin toxicity patients by ECG only with the AUC of 0.912. After adding the clinical information, the predictive capacity improved with the AUC to 0.980, although no statistical significance (Supplementary Table S2, *p*-value 0.260, 0.207, respectively).

Some consistencies and discrepancies in the prediction of digoxin toxicity exist between human experts and our AI system during competition. Sixteen out of 26 ECGs (61.5%) were correctively answered by both human experts and our AI system. Eight out of 26 ECGs (30.8%) misdiagnosed by human experts that were correctively answered by the AI demonstrated abnormal conduction and ST segment depression related to ischemia or hypokalemia. These results spotlight the strengths of our AI model in the prediction of digoxin toxicity in patients with multiple comorbidities. One out of 26 ECGs misdiagnosed by AI that was correctively answered by human experts showed bigeminal VPCs. Such evidence reminds us that bigeminal VPCs may be a potential pitfall in the prediction of digoxin toxicity by our AI system. One out of 26 ECGs incorrectly answered by both AI and human experts displayed a normal ECG presentation. As we have depicted above, and as reported by Anandhi et al., only 23.95% of patients with serum cardiac glycoside poisoning had their electrocardiographs recognized as indicating toxicity [14]. Gulsum Limon et al. reported that 11 of 28 patients with digoxin toxicity had no ECG change [13]. Thus, clinicians should still be careful during history taking, even though the electrocardiograph reveals normal findings, which were not identified by the AI system.

Importantly, our DLM had a high predictive ability of digoxin toxicity in lead I, with AUC 0.960, which is superior to the summation of the 12 leads. The characteristic presentation of digoxin toxicity demonstrates down-sloping ST segment depression in lead I. Meanwhile, Leads aVL and V6 have the second- and the third-best predictive abilities, with AUCs of 0.928 and 0.918, respectively. Studies from Saner HE et al. suggested that normal ST segments in lead I, lead aVF, V5, and V6 may exclude the presence of a >1.3 ng/mL SDC [19], which is compatible with our findings (Figure 3). Although the AUC (0.912) of our AI system using 12 leads seemed worse than that using each of the three leads, the performance regarding the summation of sensitivity and specificity using 12 leads was better than that using each of the three leads. This showed that the information of the digoxin effect was mainly contributed from lateral leads, which will require further electrophysiological research to elucidate the underlying mechanism.

To enhance the performance of our DLM, we incorporated the predictive factors of digoxin toxicity, including gender, age, hypertension, AF, and impaired renal function, in the model and found that such information increased the AUC from 0.912 to 0.987, though with no statistical significance. These results indicated that medical information may be helpful to increase the diagnostic power of our DLM in the prediction of digoxin toxicity. Interestingly, our DLM had better accuracy in detecting digoxin toxicity in patients with HF and/or without AF than those without HF and/or with AF. The enhanced sensitivity of our DLM in the prediction of digoxin toxicity in patients with HF promotes its performance. Importantly, only eight patients with HF and four patients without AF were included in the validation set for stratified analysis, which accounts for 100% of sensitivity, instead of overfitting of the data. Additionally, the low diagnostic power of our DLM in patients with AF may be caused by the irregularly irregular rhythms, which interfere with the diagnostic capacity of our DLM. These results remind us of the potential diagnostic difficulties in patients with AF and/or without HF when we apply our DLM in the detection of digoxin toxicity.

### Strengths and Limitation

By training with more than 170,000 ECGs, we set up our DLM to detect digoxin toxicity with extraordinary performance. To our knowledge, this is the first study to detect digoxin toxicity merely by AI-assisted ECG. However, several limitations should be mentioned. First, this is a single-institute and retrospective study with a limited number of digoxin toxicity cases. Second, we only included nine clinicians in the human-machine competition, which may not reflect the real-world data. However, previous studies disclosed 23.95% and 60.8% sensitivities of digoxin toxicity identified by human ECG readings [13,14], which are inferior to the clinicians included in this study, with sensitivities of 61.5% to 92.5% and specificities of 59.6% to 90.4%. Third, due to the rarity of digoxin toxicity cases, we applied the simple oversampling method to maintain the balanced cases and controls in each batch (the details are shown in Supplementary Materials). This process made the higher dependency of cases in each batch, which may hazard the model performance. Future studies should collect more digoxin toxicity ECGs to improve the DLM performance. Finally, the information regarding lifestyle factors, including smoking and alcoholism, was not included. Based on the current AI model, a prospective and large database for AI training may improve the sensitivity and specificity in the detection of digoxin toxicity.

## 5. Conclusions

We established a DLM to detect digoxin toxicity in a high-accuracy, economical, prompt, and accessible way by ECG, which may initiate rapid correction by DLM to prevent catastrophic outcomes and may serve as a convenient tool to monitor digoxin toxicity. There will be several promising applications in our DLM, including providing decision support and a high-risk alarm system for digoxin toxicity detection in the ED, being incorporated into ECG machines in ambulances or remote areas to facilitate telemedicine, and being applied to a wearable device for digoxin toxicity detection in high-risk patients. Although further study is needed to promote the capacities of our DLM and to verify its beneficial effects, our DLM provides an effective decision support system to identify digoxin toxicity in clinical practice.

## Figures and Tables

**Table 1:** Corresponding patient characteristics and laboratory results in digoxin toxicity ECGs and normal ECGs.

Patient Characteristics	Digoxin Toxicity (*n* = 61)	Normal (*n* = 177,066)	*p*-Value
**Dataset**			0.001
Training cohort	48 (78.7%)	160,868 (90.9%)	
Validation cohort	13 (21.3%)	16,198 (9.1%)	
Gender (male)	39 (63.9%)	92,684 (52.4%)	0.070
Age (years)	83.0 ± 7.0	63.4 ± 19.5	<0.001
Height (cm)	163.8 ± 7.1	162.0 ± 18.3	0.585
Weight (kg)	60.1 ± 6.5	63.8 ± 14.1	0.150
BMI (kg/m^2^)	22.5 ± 2.8	24.5 ± 8.1	0.177
DM	17 (27.9%)	47,900 (27.1%)	0.886
CAD	34 (55.7%)	43,808 (24.7%)	<0.001
Hypertension	54 (88.5%)	79,220 (44.7%)	<0.001
HF	31 (50.8%)	20,730 (11.7%)	<0.001
Lipidemia	31 (50.8%)	53,437 (30.2%)	<0.001
CKD	10 (16.4%)	23,356 (13.2%)	<0.001
COPDPP	27 (44.3%)	38,946 (22.0%)	<0.001
Pneumothorax	0 (0.0%)	893 (0.5%)	<0.001
AF	42 (68.9%)	12,497 (7.1%)	<0.001
K (mEq/L)	4.4 ± 0.8	3.9 ± 0.6	<0.001
Na (mEq/L)	134.3 ± 3.9	136.3 ± 4.8	0.002
Cl (mEq/L)	100.6 ± 6.3	102.3 ± 5.7	0.171
TCa (mg/dL)	8.8 ± 0.9	8.5 ± 0.7	0.198
FCa (mg/dL)	4.5 ± 0.2	4.4 ± 0.3	0.074
Mg (mg/dL)	2.1 ± 0.3	2.1 ± 0.3	0.833
Tro I (pg/mL)	0.1 ± 0.2	0.3 ± 3.4	0.772
BUN (mg/dL)	59.5 ± 42.9	26.0 ± 22.8	<0.001
Cr (mg/dL)	2.8 ± 2.6	1.5 ± 2.0	<0.001
eGFR	35.3 ± 19.8	76.1 ± 38.7	<0.001

BMI = Body mass index; DM = Diabetes mellitus; CAD = Coronary artery disease; HF = Heart failure; CKD = Chronic kidney disease; COPD = Chronic obstructive pulmonary disease; AF = Atrial fibrillation; K = Potassium; Na = Sodium; Cl = Chloride; TCa = Total calcium; FCa = Free calcium; Mg = Magnesium; Tro I = Troponin I; BUN = Blood urea nitrogen; Cr = Creatinine; eGFR = Estimated glomerular filtration rate. The significant level was 0.05/25 = 0.002 based on Bonferroni correction.

## Data Availability

The data presented in this study are available on request from the corresponding author.

## Supplementary Materials

The following are available online at https://www.mdpi.com/article/10.3390/ijerph18073839/s1, Table S1: Patient characteristics and laboratory results in the training and validation cohorts, Table S2: Univariable and multivariable logistic regression analyses in the validation cohort, Figure S1: The deep learning model architecture, Figure S2: Consistency analysis of answers given by the deep learning model and human experts, Figure S3: Univariable and multivariable logistic regression analyses in the training cohort. References [31,32,33,34,35,36,37,38] are cited in the supplementary materials.
