# Supplementary material for: Detecting Digoxin Toxicity by Artificial Intelligence-Assisted Electrocardiography
Source: Int J Environ Res Public Health. 2021 Apr 6;18(7):3839. doi: 10.3390/ijerph18073839 (PMC8038815; doi:10.3390/ijerph18073839)
Supplement: Supplementary file 1 [file ijerph-18-03839-s001.pdf]

International Journal of
Environmental Research and Public Health

MDPI

Supplementary Materials

Database

The primary analysis was to evaluate the performance of digoxin toxicity recognition by ECG12Net and clinicians in a human-machine competition. Receiver operating characteristic (ROC) curve and area under the curve (AUC) were applied to evaluate the competition results. Additionally, the sensitivity and specificity of digoxin toxicity recognition by the deep learning model and clinical physicians were calculated. The results of the whole validation evaluation by the deep learning model were also presented.

The secondary analyses were performed on the whole validation cohort. Lead-specific analysis was conducted to evaluate the lead-based information contributions. We tried to include more clinical information, such as patient characteristics and laboratory test results, to improve the model performance. The multivariable logistic regression model was used to integrate the deep learning model and clinical information. A series of logistic models will identify the effects of different clinical information on the performance of digoxin toxicity recognition. AUCs based on the ROC curve were applied to evaluate the change in model performance.

We split these ECGs into training (n= 48) and validation (n= 13) cohorts by date. There are no ECGs of the validation cohort belonging to patients who have ECGs used in training. Meanwhile, normal ECGs were collected from our emergency room. A total of 177,066 ECGs from 84,286 cases in the same study period were included. We also split these ECGs into training (160,868) and validation (16,198) cohorts by the same date. Patients with any laboratory record of serum digoxin concentration no less than 2 ng/mL were excluded from the normal ECGs group. All of the ECG records were collected by Philips 12-Lead ECG machines (PH080A, Philips Medical Ssytems, Andover, MA, USA). The ECG records were standardized with the 10-mm high reference pulse as 1 mV and the 5-mm width as 200 ms. Each 12-lead ECG was taken for 10 seconds, and the ECG signals were presented as a standard ECG for physician interpretation. Patient characteristics and laboratory tests were collected from our electronic medical records. The timely nearest laboratory records were assigned for each ECG. Because the ECG tests were sometimes conducted in a short time period, some ECGs from the same patients might share the same patient characteristics and laboratory records. The collected patient characteristics included gender, age, height, weight, DM, CAD, hypertension, HF, hyperlipidemia, CKD, COPD, pneumothorax, and AF. The laboratory tests included serum concentration of K, Na, Cl, total Ca, free Ca, Mg, CK, Troponin I, BUN, Cr, and serum digoxin concentration. Only the laboratory records within 24 hours were included.

Deep Learning Model Implementation

Suppose that a standard 12-lead ECG signal comprises 12 sequences of N numbers (n = 1250 in our database). To detect the digoxin toxicity, ECG signal sequence X = [x1,1, x1,2, …, x1,n; x2,1, x2,2, …, x2,n; …; x12,1, x12,2, …, x12,n] is used as the input, and the output is one-hot encodes of digoxin toxicity categories (digoxin toxicity and non-digoxin toxicity). For example, a label of digoxin toxicity is encoded as [1, 0], and a label of non-digoxin toxicity is encoded as [0, 1], respectively. Each output label corresponds to a segment of the input. Because the ECG information is mostly provided by morphologic changes with shift invariance, convolutional layers with weight sharing were used to adapt to this situation and reduce the hazard of overfitting. We therefore developed a 12-channel sequence-to-sequence model to conduct this task as a revision of DenseNet [31]. The complete architecture of ECG12Net is shown in Figure S1.

We defined a “dense unit” as a neural combination, as follows: (1) a batch normalization layer to normalize input data [32], (2) a rectified linear unit (ReLU) layer for nonlinearization [33], (3) a 1 × 1 convolution layer with 4K filters to reduce the dimensions of the data, (4) a batch normalization layer for normalization, (5) a ReLU layer for non-

linearization, and (6) a 3 × 1 convolution layer with 4K filters to extract features, (7) a batch normalization layer for normalization, (8) a ReLU layer for non-linearization, and (9) a 1 × 1 convolution layer with K filters to extract features. K is a model constant, which was set at 32 in all our experiments. After using a dense unit to extract features, we used the dense connectivity resulting from direct connections from any layer to all subsequent layers to build a "dense block." We designed a model with five dense blocks comprising 3, 3, 6, 6, and 3 dense units, respectively. Dense blocks cannot be concatenated when the size of feature maps change. Thus, a pooling block was used to concatenate each dense block for down-sampling in our architecture. This block included a dense unit with 2 × 1 stride and an average pooling layer with a 2 × 1 kernel size and stride, which was used for down-sampling [34]. Each dense block was concatenated by the pooling block to integrate the features of the previous blocks.

A length of 864 numeral sequences was used as the input in our experiment. We designed an ECG lead block with 80 trainable layers, whose architecture is shown in Figure S1A. The input data were passed through a batch normalization layer, followed by a convolution layer, another batch normalization layer, a ReLU layer, and a pooling layer. The initial convolution layer comprised K convolution filters with a kernel size of 7 × 1 and a stride of 2 × 1. Next, the data were passed through a series of dense blocks and a pooling block, resulting in a 16 × 1 × 864 array. A ReLU layer, a batch normalization layer, and a global pooling layer were followed by the last dense block. Finally, a fully connected layer with k output was created for follow-up use. Where k is the number of categories, and it is equal to 3 in the first MI detection model and 4 in the second location analysis model of STEMI, respectively. This ECG lead block was used to extract 864 features from each ECG lead, making a basic output prediction based on each lead. Figure S1B shows how ECG12Net integrated all the information from the ECG leads to make an overall prediction. ECG12Net comprised 12 ECG lead blocks corresponding to lead sequences. We designed an attention mechanism based on a hierarchical attention network to concatenate these blocks, increasing the interpretive power of ECG12Net [35]. The attention block comprised a batch normalization layer followed by a fully connected layer and then two combinations of a batch normalization layer, a ReLU layer, and a fully connected layer. The first and second fully connected layers each contained $8/k$ neurons. Attention scores were calculated for each ECG lead and then integrated for standardization by a linear output layer. The standardized attention scores were used to weight the 12 ECG lead outputs by simple multiplication. The 12 weighted outputs were summed and converted into a softmax output layer to give the final prediction value. The above model using ECG information only was named ECG12Net, which contained 82 trainable layers. The m-log-loss function was used to calculate model loss. A dropout layer [36] was added in the only fully connected layer, and the dropout rate was set to 0.5.

Training Details

The 12-lead ECG signal sequences were first trained separately by the 12 ECG leads. Due to uneven distribution of digoxin toxicity cases and controls, an oversampling process was implemented to improve performance by ensuring that rare samples were adequately recognized [34,35]. We sampled 18 ECGs from digoxin toxicity cases and 18 ECGs from controls in each batch. This process sufficiently considered rare digoxin toxicity cases so as not to be skewed by the overwhelming number of controls. We used the software package MXNet version 1.3.0 [36] to implement ECG12Net. The settings used for the training model were as follows: (1) Adam optimizer with standard parameters (β_1 = 0:9 and β_2 = 0:999) and a batch size of 36 for optimization; (2) initial learning rate set at 0.001 and lowered by 10 three times when validation loss plateaued after an epoch; and (3) a weight decay of 10^{-4} [37]. Because the sampling rate of our machine is 500 Hz, our 12-lead ECG signal includes 12 numeral sequences with 5,000 digits. However, the standard input format of ECG12Net is a length of 1024 numeric sequences. We randomly cropped a length of 1024 sequences as input in the training process. The initial weights of the digoxin

toxicity recognition model were based on the transfer learning from the potassium concentration prediction model [33].

The patients in the training cohort were divided into five subgroups, and we used the 5-fold cross-validation which used four of them as training subset and 1 of them as tuning subset in each fold. In other words, we totally trained five DLMs, and the final prediction in validation cohorts was generated by the average of them. During the inference stage, the nine overlapped a length of 1024 sequences based on interval sampling (X_{1} to X_{1024}, X_{498} to X_{1521}, X_{995} to X_{2018}, X_{1492} to X_{2515}, X_{1989} to X_{3012}, X_{2486} to X_{3509}, X_{2983} to X_{4006}, X_{3480} to X_{4503}, and X_{3977} to X_{5000}) and were used to generate prediction and averaged as the final prediction. Finally, a total of 45 standardized probabilities predicted via five DLMs and nine sequences were generated for each ECG in the validation cohort, and the average of them was used as the final probability. For deciding the cut-point for the final probability, we applied the receiver operating characteristic (ROC) curve to each tuning subset. A total of five cut-points for maximizing the sum of sensitivity and specificity were selected in each subset, and the mean of them was used as the cut-point for final probability.

A previous study reported severe overfitting in an atrial fibrillation detection task and suggested a series of data augmentations to improve model performance [38]. In the current study, the problem of overfitting was due to the large number of parameters in the deep learning architecture (~3 million trainable parameters) relative to the sample size. The first step in tackling this issue was to resize sequence length by adjusting heart rate. We randomly resampled a broader range of heart rates in a uniform distribution from 0.8*HR* to 1.2*HR*, where *HR* is the original heart rate for each sample. The second step was to randomly crop a length of 1024 sequences as input. The third step was to add a random variable drawn from a Gaussian distribution with a mean of 0 and a standard deviation of 0.1. Fourth, time points were selected uniformly and at random, and the ECG signal values in a 50 ms vicinity of these points were set at 0. This method is called dropout burst [38]. Finally, we set six random ECG lead sequences to 0 in the combined training step. We observed that the final deep learning model only used information from a few ECG leads to make a prediction, which inferred that the model had ceased to learn features from other ECG leads because it had perfectly predicted all the data in the training set. This approach forced the deep learning model to learn all the abnormal ECG leads.

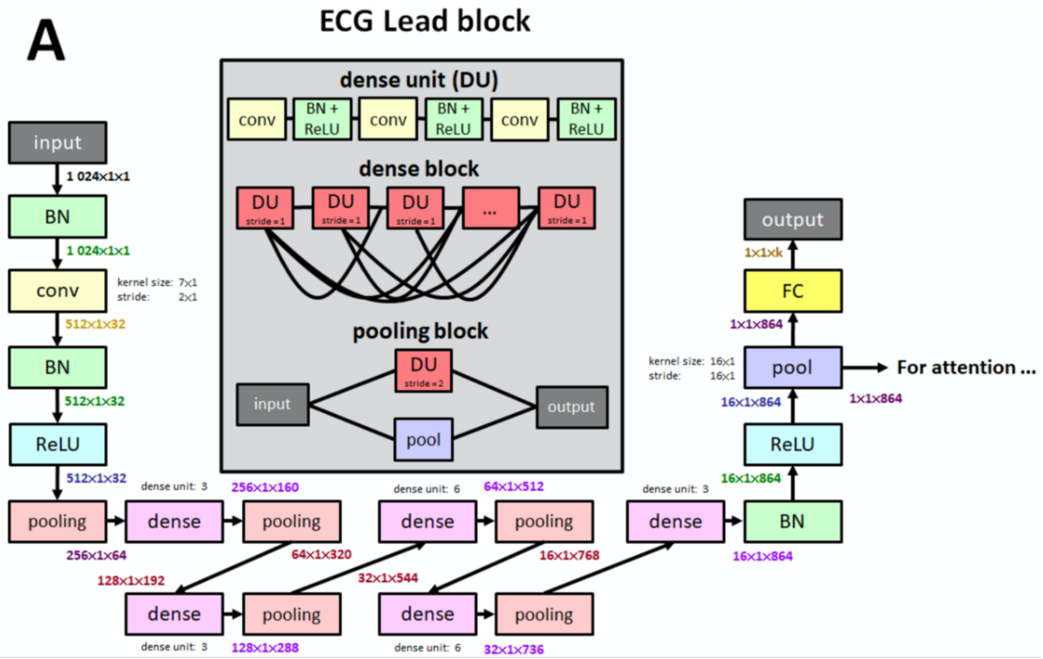

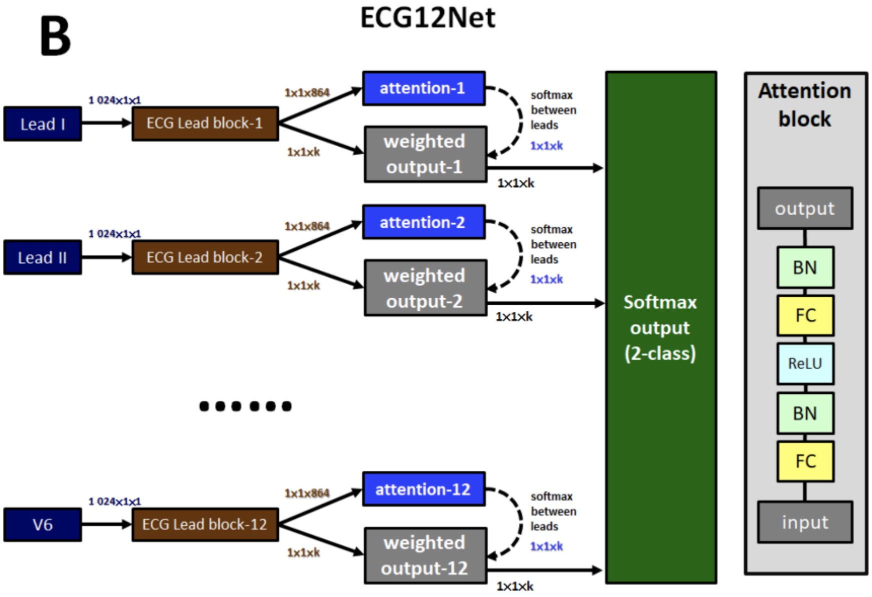

Figure S1. The deep learning model architecture. The training model is shown as above and described in the supplementary text. BN = Batch Normalization; conv = convolution layer; ReLU = Rectified Linear Units; dense = dense unit; FC = fully connected layer. (**A**) All digoxin toxicity ECGs, ranking from lower disease score to higher disease score by AI analysis. (**B**) Selected normal ECGs, ranking from higher disease score to lower disease score.

Table S1. Patient characteristics and laboratory results in the training and validation cohorts.

Patients Characteristics	Training Cohort (*n* = 160,916)	Validation Cohort (*n* = 16,211)	*p*-Value
**Gender (male)**	84,219 (52.3%)	8504 (52.5%)	0.765
**Age (years)**	63.2 ± 19.4	64.8 ± 20.0	<0.001
**Height (cm)**	162.0 ± 18.6	161.2 ± 8.8	0.032
**Weight (kg)**	63.8 ± 14.1	64.0 ± 13.6	0.548
**BMI (kg/m²)**	24.5 ± 8.2	24.6 ± 4.8	0.570
**DM**	43,200 (26.9%)	4717 (29.1%)	<0.001
**CAD**	38,742 (24.1%)	5100 (31.5%)	<0.001
**Hypertension**	70,835 (44.0%)	8439 (52.1%)	<0.001
**HF**	18,544 (11.5%)	2217 (13.7%)	<0.001
**Lipidemia**	48,370 (30.1%)	5098 (31.5%)	<0.001
**CKD**	21,543 (13.4%)	1823 (11.2%)	<0.001
**COPD**	34,876 (21.7%)	4097 (25.3%)	<0.001
**Pneumothorax**	834 (0.5%)	59 (0.4%)	0.008
**AF**	11,373 (7.1%)	1166 (7.2%)	0.553
**K**	3.9 ± 0.6	3.9 ± 0.6	<0.001
**Na**	136.3 ± 4.8	136.1 ± 5.0	<0.001
**Cl**	102.4 ± 5.7	101.3 ± 7.7	<0.001
**TCa**	8.6 ± 0.7	8.4 ± 0.7	<0.001
**FCa**	4.4 ± 0.3	4.4 ± 0.3	<0.001
**Mg**	2.1 ± 0.3	2.1 ± 0.4	0.002
**Tro I**	0.3 ± 3.4	0.2 ± 3.2	0.143
**BUN**	26.2 ± 23.0	24.1 ± 20.3	<0.001
**Cr**	1.6 ± 2.0	1.4 ± 1.7	<0.001
**eGFR**	76.2 ± 39.0	74.1 ± 35.9	<0.001

Numbers of missing values: Gender: 26; Age: 38; Height: 124,670; Weight: 124,670; BMI = Body mass index: 124,670; DM = Diabetes mellitus: 26; CAD = Coronary artery disease: 26;

Hypertension: 26; HF = Heart failure: 26; Lipidemia: 26; CKD = Chronic kidney disease: 26; COPD = Chronic obstructive pulmonary disease: 26; Pneumothorax: 26; AF = Atrial fibrillation: 26; K = Potassium: 3096; Na = Sodium: 3783; Cl = Chloride: 113,603; TCa = Total calcium: 153,425; FCa = Free calcium: 161,379; Mg = Magnesium: 159,463; Tro I = Troponin I: 27,321; BUN = Blood urea nitrogen: 89,508; Cr = Creatinine: 3918; eGFR = Estimated glomerular filtration rate: 3956. The significant level was 0.05/24 = 0.002 based on Bonferroni correction.

Table S2. Univariable and multivariable logistic regression analyses in the validation cohort.

Patient Characteristics	AI Model		Integration Model 1		Integration Model 2	
	OR (95% CI)	*p*-Value	OR (95% CI)	*p*-Value	OR (95% CI)	*p*-Value
**Disease score**	1.09 (1.07–1.11)	<0.001	1.08 (1.06–1.11)	<0.001	1.08 (1.06–1.11)	<0.001
**Gender (male)**			0.44 (0.13–1.53)	0.199	0.48 (0.13–1.75)	0.263
**Age (years)**			1.01 (0.96–1.06)	0.668	1.00 (0.95–1.06)	0.995
**Hypertension**			0.70 (0.18–2.72)	0.606	0.54 (0.13–2.20)	0.386
**AF**			10.74 (3.01–38.32)	<0.001	16.89 (4.13–69.11)	<0.001
**eGFR**					0.96 (0.94–0.99)	0.005
**AUC**	0.912		0.980		0.987	
***p*-Value (AUC)**	NA		0.260		0.207	

The significant level was 0.05/6 = 0.008 based on Bonferroni correction.

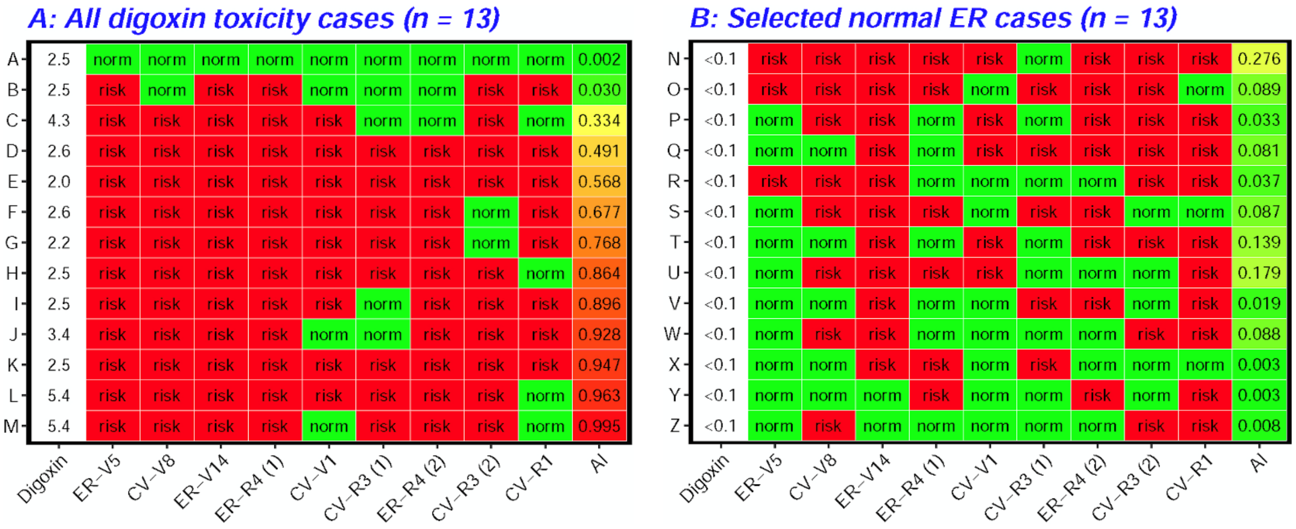

Figure S2. Consistency analysis of answers given by the deep learning model and human experts: the answers given by our physicians were "risk" and "norm", corresponding to potential digoxin toxicity and normal ECGs, respectively. The numbers shown in the last column are the probabilities given by the AI. The positive cut-off of points is 0.334 in this analysis based on previous ROC curves, and the colors green, yellow, and red represent the low, middle, and high risk levels identified by the AI, respectively. There are 4 emergency physicians and 5 cardiologists participating in this competition. The emergency physicians include 2 residents and 2 attending physicians, indicated as ER-R4(1), ER-R4(2), ER-V5, and ER-V14. The cardiologists include 3 residents and 2 attending physicians, indicated as CV-R1, CV-R3(1), CV-R3(2), CV-V1, and CV-V8. The sensitivities/specificities were 76.9%/69.2%, 92.3%/63.5%, 84.6%/80.8%, 76.9%/75.0%, 61.5%/65.4%, 69.2%/88.5%, 92.3%/59.6%, 61.5%/90.4%, and 92.3%/90.4% in ER-R4 (2), ER-R4 (1), CV-V8, CV-R3 (2), CV-R1, CV-V1, ER-V14, CV-R3 (1), and ER-V5, respectively. (**A**) All digoxin toxicity cases. (**B**) Selected normal ER cases.

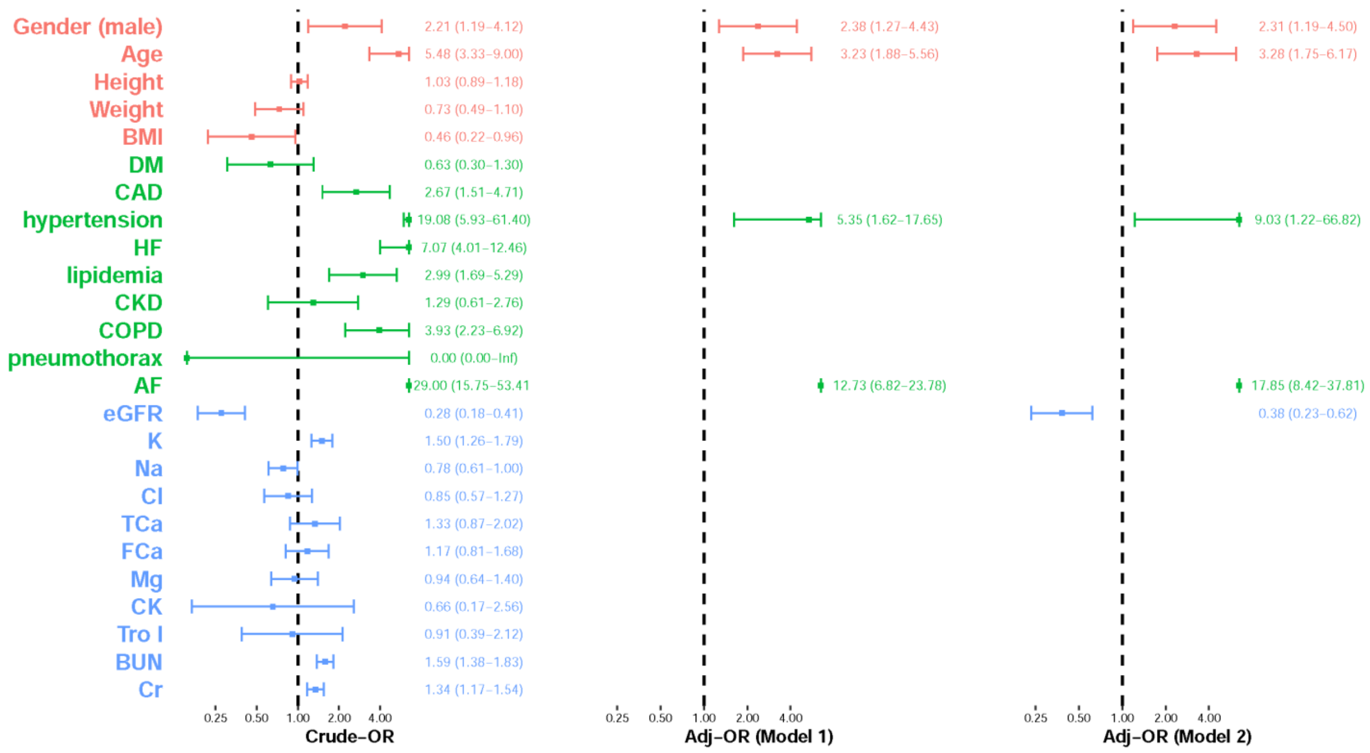

Figure S3. Univariable and multivariable logistic regression analyses in the training cohort: model 1 only included the significant patient demographics and disease history, and model 2 additionally included other significant laboratory tests. All other variables not included in the multivariable analysis were not significantly associated with digoxin toxicity. The continuous variables are standardized by mean and standard deviation; therefore, the units of each continuous variable were 1 standard deviation.

References

1. Huang, G.; Liu, Z.; Van Der Maaten, L.; Weinberger, K.Q. Densely Connected Convolutional Networks. In Proceedings of the 2017 IEEE Conference on Computer Vision and Pattern Recognition (CVPR), Honolulu, HI, USA, 21–26 July 2017; pp. 2261–2269.
2. Ioffe, S.; C. Szegedy. Batch normalization: Accelerating deep network training by reducing internal covariate shift. In 32nd International Conference on International Conference on Machine Learning, Lille, France, 6–11 July 2015.
3. Glorot, X.; Bordes, A.; Bengio, Y. Deep Sparse Rectifier Neural Networks. In Proceedings of the 14th International Conference on Artificial Intelligence and Statistics, Fort Lauderdale, FL, USA, 11–13 April 2011.
4. LeCun, Y.; Bottou, L.; Bengio, Y.; Haffner, P. Gradient-based learning applied to document recognition. In *Proceedings of the Proceedings of the IEEE* **1998**, *86*, 2278–2324.
5. Yang, Z.; Yang, D.; Dyer, C.; He, X.; Smola, A.; Hovy, E. Hierarchical Attention Networks for Document Classification. In Proceedings of the Proceedings of the 2016 Conference of the North American Chapter of the Association for Computational Linguistics: Human Language Technologies, San Diego, CA, USA, June 2016.
6. Srivastava, N.; Hinton G.; Krizhevsky A.; Sutskever I.; Salakhutdinov R. Dropout: a simple way to prevent neural networks from overfitting. *J. Mach. Learn. Res.* **2014**, *15*, 1929–1958.
7. Gross, S.; Wilber, M. Training and investigating residual nets. Facebook AI Research, CA. Available online: http://torch.ch/blog/2016/02/04/resnets.html (accessed on 6 April 2021).
8. Zihlmann, M.; Perekrestenko, D.; Tschannen, M. Convolutional Recurrent Neural Networks for Electrocardiogram Clas-sification. *arXiv* **2017**, arXiv:1710.06122. Available online: https://arxiv.org/abs/1710.06122 (accessed on 6 April 2021).
